# Wrist-Worn Activity Trackers in Laboratory and Free-Living Settings for Patients With Chronic Pain: Criterion Validity Study

**DOI:** 10.2196/24806

**Published:** 2021-01-12

**Authors:** Veronica Sjöberg, Jens Westergren, Andreas Monnier, Riccardo Lo Martire, Maria Hagströmer, Björn Olov Äng, Linda Vixner

**Affiliations:** 1 School of Education, Health and Social Studies Dalarna University Falun Sweden; 2 Military Academy Karlberg Swedish Armed Forces Solna Sweden; 3 Department of Neurobiology, Care Sciences and Society Division of Physiotherapy Karolinska Institutet Huddinge Sweden; 4 Academic Primary Health Care Centre Region Stockholm Stockholm Sweden; 5 Center for Clinical Research Dalarna Uppsala University, Region Dalarna Falun Sweden

**Keywords:** chronic pain, energy expenditure, heart rate, physical activity, step count, validity, wearable devices, wearable, pain, rehabilitation

## Abstract

**Background:**

Physical activity is evidently a crucial part of the rehabilitation process for patients with chronic pain. Modern wrist-worn activity tracking devices seemingly have a great potential to provide objective feedback and assist in the adoption of healthy physical activity behavior by supplying data of energy expenditure expressed as metabolic equivalent of task units (MET). However, no studies of any wrist-worn activity tracking devices’ have examined criterion validity in estimating energy expenditure, heart rate, or step count in patients with chronic pain.

**Objective:**

The aim was to determine the criterion validity of wrist-worn activity tracking devices for estimations of energy expenditure, heart rate, and step count in a controlled laboratory setting and free-living settings for patients with chronic pain.

**Methods:**

In this combined laboratory and field validation study, energy expenditure, heart rate, and step count were simultaneously estimated by a wrist-worn activity tracker (Fitbit Versa), indirect calorimetry (Jaeger Oxycon Pro), and a research-grade hip-worn accelerometer (ActiGraph GT3X) during treadmill walking at 3 speeds (3.0 km/h, 4.5 km/h, and 6.0 km/h) in the laboratory setting. Energy expenditure and step count were also estimated by the wrist-worn activity tracker in free-living settings for 72 hours. The criterion validity of each measure was determined using intraclass and Spearman correlation, Bland-Altman plots, and mean absolute percentage error. An analysis of variance was used to determine whether there were any significant systematic differences between estimations.

**Results:**

A total of 42 patients (age: 25-66 years; male: 10/42, 24%; female: 32/42, 76%), living with chronic pain (duration, in years: mean 9, SD 6.72) were included. At baseline, their mean pain intensity was 3.5 (SD 1.1) out of 6 (Multidimensional Pain Inventory, Swedish version). Results showed that the wrist-worn activity tracking device (Fitbit Versa) systematically overestimated energy expenditure when compared to the criterion standard (Jaeger Oxycon Pro) and the relative criterion standard (ActiGraph GT3X). Poor agreement and poor correlation were shown between Fitbit Versa and both Jaeger Oxycon Pro and ActiGraph GT3X for estimated energy expenditure at all treadmill speeds. Estimations of heart rate demonstrated poor to fair agreement during laboratory-based treadmill walks. For step count, the wrist-worn devices showed fair agreement and fair correlation at most treadmill speeds. In free-living settings; however, the agreement for step count between the wrist-worn device and waist-worn accelerometer was good, and the correlation was excellent.

**Conclusions:**

The wrist-worn device systematically overestimated energy expenditure and showed poor agreement and correlation compared to the criterion standard (Jaeger Oxycon Pro) and the relative criterion standard (ActiGraph GT3X), which needs to be considered when used clinically. Step count measured with a wrist-worn device, however, seemed to be a valid estimation, suggesting that future guidelines could include such variables in this group with chronic pain.

## Introduction

Chronic pain is defined as “pain that persists past normal healing time and hence lacks the acute warning function of physiological nociception” [1,2] and is a leading major public health problem internationally due to its effects on physical, social, and emotional functions [3]. Physical activity is a central part of chronic pain rehabilitation due to the evident health benefits, which include improved cardiovascular health, prolonged lifespan [4,5], positive effects on pain intensity, health-related quality of life, and both physical and psychological functions [6]. The American Heart Association has provided guidelines regarding sufficient weekly amounts of physical activity to reap health benefits for a healthy population, as well as for populations with chronic conditions [4,7]. For patients with chronic pain, recommendations are to spend ≥150 minutes/week engaged in moderate-to-vigorous physical activity (MVPA). Moderate physical activity is defined as equal to or more than 3 and less than 6 metabolic equivalent task units (MET) [8]. One MET is defined as a resting metabolic rate obtained when quietly seated [8]. Despite clear guidelines, it seems that inadequate physical activity levels are common among patients with chronic pain, which can lead to an increased risk of physical and mental illness [5]. In rehabilitation settings, objective estimations of physical activity are rarely used. Instead, subjective measures are common practice due to their high degree of acceptance, cost effectiveness, and relatively low administrative burden [9]. However, despite its perceived benefits, subjective estimations of physical activity domains have estimation biases, such as recall bias and reactivity bias [9]. Several studies [10-14] have indicated the potential of wrist-worn activity tracking devices as tools that can facilitate behavior change and increase the degree to which patients follow individually modulated physical activity levels designed to improve health. Wearable devices for physical activity tracking have received increased interest from both the research community and consumers aiming to quantify domains of physical activity (eg, frequency and duration) in order to optimize health behaviors [10,15,16]; however, before the clinical use of these devices can be introduced, the validity of each device needs to be established [17]. In the past decade, there has been an increasing number of studies [18-22] assessing the validity of wrist-worn tracking devices that measure energy expenditure by comparison to a criterion standard such as indirect calorimetry or accelerometry. The majority of these validation studies were conducted among healthy adult participants [17,23], with studies reporting somewhat conflicting findings—both overestimation [20,23] and underestimation [19,24,25] with Fitbit devices were reported. In a recent systematic review [23] investigating the accuracy of Fitbit devices, it was reported that 49% (43 of 88 comparisons) overestimated energy expenditure, particularly during physical activity. In an earlier systematic review of the field, Evenson et al [17] reported a high validity of different brands of wearable activity tracking devices regarding step count when compared to various criterion standards made in laboratory settings [26,27]. Regarding the validity of heart rate estimations from by wrist-worn activity tracking devices, one study [28] have shown that the agreement between true rate and the estimated rate made by a wrist-worn device is higher during rest than during MVPA in healthy subjects. To our knowledge, there has been no prior research examining wrist-worn activity tracking device criterion validity in estimating energy expenditure (using MET), step count, or heart rate among patients with chronic pain. This lack of research constitutes a substantial knowledge gap given how important it is for patients with chronic pain to achieve adequate amounts of weekly physical activity. Therefore, the aim of this study was to evaluate the criterion validity of each of these measures estimated by a wrist-worn activity tracking device for patients with chronic musculoskeletal pain in both laboratory and free-living settings.

## Methods

### Study Design

We conducted a laboratory and field validation study. Data were collected between March 2019 to June 2020 (Health and Sports laboratory, Dalarna University). The sample size calculation was based on intraclass correlation (ICC), the primary statistic in the study. In order to achieve 80% power to detect an ICC of 0.80 (excellent agreement) with a 95% distribution (lower limit 0.6), calculation based on published recommendations [29] showed a requirement of 26 to 49 participants. This study was approved by Swedish Ethical Review authority (registration number 2018-307).

### Recruitment and Study Sample

The inclusion criteria were adult age (between 18 and 67 years), with chronic (>3 months) musculoskeletal (neck or low back) pain or widespread pain, currently undergoing assessment or treatment (for chronic pain) in a primary or specialized health care clinic, and having the ability to understand information in Swedish. The exclusion criteria were having given birth within the previous 3 months, pregnant in the second or third trimester, requiring a walking aid indoors, currently undergoing heart assessment or investigation, with pain caused by malignancy or systematic disease, or having a known allergy to plaster or adhesive tape. Participants were recruited from 8 primary and specialized health care clinics in Region Dalarna. Patients who matched the study criteria (age, duration of pain, language) were asked by clinicians for consent to be contacted by a study representative, who conducted additional screening for eligibility. At the test site, for safety reasons, all participants declared whether they had been diagnosed with or experienced a heart condition, chest pain, dizziness, high or low blood pressure, any respiratory disorder, or diabetes before any tests were performed. Participants’ height and weight were manually measured using a stadiometer (Holtain Limited) and a weighing scale (Sartorius AG). A self-rated questionnaire captured date of birth; biological sex; education level; work status; years lived with pain; and pharmaceutical, caffeine, and nicotine consumption in the previous 24 hours. Participants also completed the Swedish National Board of Health and Welfare’s questionnaire on physical activity level (minutes per week spent in exercise and in physical activity) [30,31]. In addition, participants completed the Multidimensional Pain Inventory (in Swedish) to describe psychosocial and behavioral consequences of pain [32].

### Equipment

A wrist-worn activity tracker (Fitbit Versa, Fitbit Inc), chosen for its high degree of user-friendliness, because it can be used with web or smartphone apps, and it is suitable for water activities. The Fitbit Versa estimates movement (eg, active minutes) using a triaxial accelerometer and MET/minutes based on a combination of basal metabolic rate (adjusted for sex, age, height, and weight), accelerometry-based activity counts, and heart rate measured through optical sensors [33,34].

The criterion standard (gold standard) for energy expenditure in our laboratory setting was indirect calorimetry from pulmonary gas exchange. Oxygen uptake (VO_2_) and carbon dioxide production (VCO_2_) was measured using a mixing-chamber system (Jaeger Oxycon Pro) that measures respiratory gas exchange through a mouthpiece and tube [35]. Jaeger Oxycon Pro provides an assessment of resting energy expenditure and activity-related energy expenditure based on type and amount of substrate oxidized and the amount of energy produced by biological oxidation—MET values are based on the equation: 1 MET = 3.5 mL/min/kg VO_2_ [8]. Before the start of the testing protocol, ambient conditions were recorded, and automatic volume and gas calibration was performed using a high-precision gas mixture (Air Liquide AB). The Jaeger Oxycon Pro has been validated by comparison to the Douglas bag-method and has been found a reliable criterion standard for indirect calorimetry [36]. Real-time VO_2_ and heart rate data were recorded throughout the entire laboratory protocol.

The relative criterion standard was a research-grade hip-worn accelerometer: ActiGraph GT3X-BL (ActiGraph LLC) and appurtenant software Actilife (version 6.13.3; ActiGraph LLC). The ActiGraph GT3X is a research-based triaxial accelerometer commonly used as a criterion standard both in free-living and in laboratory settings, within various populations as it is a valid and reliable tool to quantify physical activity [11,37,38].

### Procedures

According to current guidelines [39,40], in investigations aiming to evaluate the criterion validity of a wrist-worn activity tracker, data collection should be conducted in laboratory and free-living settings. In the laboratory setting, energy expenditure data were concurrently collected from Jaeger Oxycon Pro and Fitbit Versa during rest (sitting quietly seated for 10 minutes) and during treadmill walking (18 minutes). Heart rate data were also collected with a chest band (Polar HR10). Step count was estimated by ActiGraph GT3X and Fitbit Versa. The last 2 minutes of each activity (rest, treadmill speed) was included in data analysis providing data during a steady state environment [41]. During rest, participants were seated (wearing the facemask with tube) in an inclined chair with supported arms, under a blanket to avoid feeling cold. The room temperature was set at 20 °C, and the laboratory was kept quiet during the resting period. The treadmill walk protocol consisted of 6 minutes at each speed of 3.0 km/h, 4.5 km/h, and 6.0 km/h. At the end of each 6 minutes, participants rated perceived exertion according to (Borg Rating of Perceived Exertion, rating from 6-20) [42], and after the third final speed, pain intensity was also assessed using a visual analog scale (0 mm to 100 mm) [43]. In the free-living setting, step count was concurrently estimated by Fitbit Versa and ActiGraph GT3X for the subsequent 72 hours after the laboratory testing [39]. Participants were instructed to wear the devices simultaneously for at least 10 hours each day, to remove the devices for sleeping, showering, and bathing, and to record their wear-time in a logbook. Data collection started once participants left the laboratory. A schematic overview for the study procedure is shown in Table 1.

**Table 1:** A schematic overview of the study procedure, measurements, and outcomes in both settings.

Activity		Duration	Instruments and devices	Outcomes
**Laboratory setting**				
	Baseline measurements	N/A^a^	Sartorius weighting scale	Weight
			Holtain Stadiometer	Height
	Questionnaires	N/A	Multidimensional Pain Inventory	Personal characteristics Pain characteristics
			National Board of Health and Welfare questions for physical activity level	Physical activity level
	Seated rest measurements	10 minutes	Jaeger Oxycon Pro	Energy expenditure, heart rate
			ActiGraph GT3X	Energy expenditure
			Fitbit Versa	Energy expenditure, heart rate
	Treadmill walk measurements	18 minutes (6 minutes at 3.0, 4.5, 6.0 km/h each)	Jaeger Oxycon Pro	Energy expenditure, heart rate
			ActiGraph GT3X	Energy expenditure, step count
			Fitbit Versa	Energy expenditure, heart rate, step count
			Borg´s RPE scale (6-20)	Perceived exertion
			Visual Analogue Scale (0-100)	Pain intensity post–treadmill walk
**Free-living setting**				
	Free-living activities	72 hours	ActiGraph GT3X	Energy expenditure, step count
			Fitbit Versa	Energy expenditure, step count
			Logbook	Wear-time

^a^N/A: not applicable.

### Experimental Measurement

The Fitbit Versa was initialized, and participants’ age, height, length, and biological sex were registered. The device was synchronized to its app (Fitbit Dashboard) and fitted on participants’ nondominant wrist according to the manufacturer’s recommendations. To retrieve data (energy expenditure, step count, heart rate) we deployed a web-based application programming interface [44] with assistance from an experienced computer programmer. Through such script, Fitbit allows users to download defined data by minute resolution. After the devices were returned, they were resynchronized before data was downloaded.

### Criterion Standard

Participants’ biological sex, height, and weight were entered into the software. Data (energy expenditure, heart rate) retrieved from Jaeger Oxycon Pro and Polar HR10 were manually aggregated to minute resolution (from 15 s to 60 s) to correspond with Fitbit Versa and ActiGraph GT3X data output.

### Relative Criterion Measurement

ActiGraph GT3X was initialized at the 30 Hz sample rate and participants’ date of birth, height, length, and biological sex were entered. The device was fitted on participants’ waists, to the right of the spine, using an elasticated belt. Data (counts per axis, step count) were downloaded in epochs of 60 seconds, which is commonly used in corresponding research [45]. After download, we applied a cut-off (combining the Work-Energy Theorem and the Freedson equation) in Actilife software (version 6.13.4; ActiGraph LLC) that combines to calculate energy expenditure [46]. Actilife calculates MET values based on brand-specific activity counts and chosen cut points. We applied the Freedson cut-point to score MET per minute [47].

### Data Management and Statistics

Frequency analysis of data was performed to identify potential errors. Manual checking of random samples (20% of the data)was carried out and deemed satisfactory with <3% error rate. Descriptive statistics were used to describe participant characteristics. The Shapiro-Wilk test was used to determine whether data were normally distributed. The criterion validity was determined through assessment of agreement as well as assessment of correlation between estimations and measurements of primary outcomes energy expenditure, heart rate, and step count in laboratory and free-living [39,40]. Agreement was assessed with ICC coefficient analysis (2-way random, average measures, 95% CI, absolute agreement) [48,49]. An ICC below 0.4 was considered poor, an ICC between 0.4 and 0.59 fair, an ICC between 0.6 and 0.74 good, and an ICC above 0.75 was considered as excellent [50]. Analysis of variance (ANOVA) was used to determine any significant systematic differences between estimations. To visualize the absolute, unscaled agreement [48,51], Bland-Altman plots with 95% CI (ie, limits of agreement, LOA) were calculated. Values beyond ±3 SD were identified as outliers and were excluded from analysis after sensitivity analysis. To determine correlation between estimations of energy expenditure, step count, and heart rate, Spearman (ρ) bivariate correlation analysis was used, and ρ<0.2 was considered poor, 0.2≤ρ<0.6 was considered fair, 0.6≤ρ<0.8 was considered moderate, 0.8≤ρ<0.9 was considered very strong, 0.9≤ρ<1 was considered perfect [52,53]. In addition, mean absolute percentage error (MAPE) were calculated as a measure of accuracy for both measured energy expenditure, steps, and heart rate as the mean difference between estimations of the wrist-worn activity tracker and estimations of the criterion measurement (Jaeger Oxycon Pro or ActiGraph GT3X) multiplied by 100, divided by the mean of the criterion measurement (Jaeger Oxycon Pro or ActiGraph GT3X) [27]. An MAPE value <1% was acceptable in the laboratory context [28,54] and a MAPE <10% of the criterion value was considered an acceptable rate of error in the free-living setting [9]. Missing data analysis was performed as recommended by Fox-Wasylyshyn [55] to evaluate any significant association between missing data and participant characteristics at baseline. Our predetermined significance level for *P* values was .05

## Results

### Participants

A total of 42 patients (female: 32/42, 76%; male: 10/42, 24%) participated in the study, but only 41 participants completed the protocol due to the malfunction of 1 device. The participants’ mean age was 43.8 years (SD 11.8). Participants’ mean BMI was 29.4 (SD 5.8), 66% of participants (27/41) were working/studying at the time of the study, and 49% (20/41) stated that they were physically active 150 minutes/week or more (Table 2). Most participants (36/41, 88%) completed all 3 treadmill speeds, while the remaining participants (5/41, 12%) discontinued the treadmill test at the highest speed due to high physical exertion or increased pain. Missing analysis revealed 1 significant result—all participants who discontinued the treadmill walk at the highest speed reported being physically active <150 minutes/week at baseline, while 44% (16/36) among those who completed all 3 treadmill speeds rated <150 minutes/week (*P=*.05). The mean ratings of perceived exertion at the end of each treadmill walk were 9 (SD 2), 12 (SD 2), and 14 (SD 2) for 3.0 km/h, 4.5 km/h, 6.0 km/h. Pain intensity ranged from 1 mm to 96 mm, mean 43 mm (SD 29 mm) on the visual analog scale after completion of the treadmill walk. Within the 24 hours prior to testing, 15 of the 41 participants (37%) used analgesics, and 2 (<5%) used beta blockers. Because 3 participants did not return their logbooks, data from 38 participants were included in the free-living analyses. The mean wear-time of the devices during the free-living period was 31 hours and 23 minutes (SD 6 hours and 21 minutes).

**Table 2:** Personal and pain characteristics of participants.

Characteristic			Value (n=41)	
**Demographic characteristic**				
	**Sex, n (%)**			
		Female		31 (76)
		Male		10 (24)
	Age (years), mean (SD)		43.8 (11.8)	
	BMI, mean (SD)		29.4 (5.8)	
	**Education level, n (%)**			
		Elementary		1 (2)
		Secondary		28 (68)
		University		12 (29)
		Other unspecified		1 (2)
	**Working/studying, n (%)**			
		Yes		27 (66)
		No		14 (34)
	**Treatment, n (%)**			
		Primary health care		33 (80)
		Specialized		8 (20)
**Pain characteristics**				
	**Multidimensional pain inventory, Swedish version (0-6), part 1, mean (SD)**			
		Pain intensity		3.6 (1.1)
		Pain interference		3.7 (0.8)
		Life control		3.4 (1.0)
		Affective distress		2.9 (0.9)
		Social support		3.6 (1.3)
	Number of pain locations (0-36)		14.0 (9.5)	
	**Years lived with pain, n (%)**			
		0-5^a^		20 (49)
		6-10		5 (12)
		<10		15 (37)
	**Pharmaceutical consumption last 24 hours, n (%)**			
		Analgesics		15 (37)
		Beta blockers, n (%)		2 (<5)
**Physical activity level^b^**				
	**Exercise^c^** **(minutes/week), n (%)**			
		0-30		15 (37)
		31-90		11 (27)
		91-120		11 (27)
		>120		4 (10)
	**Physical activity^d^ (minutes/week), n (%)**			
		0-60		8 (20)
		61-150		13 (32)
		151-300		7 (17)
		>300		13 (32)

^a^All participants had experienced pain >3 months.

^b^National Board of Health and Welfare's questions for physical activity level.

^c^Structured physical activity requiring physical effort and aims to improve health and fitness.

^d^Any bodily movement produced by skeletal muscles that requires energy expenditure.

### Criterion Validity

**Figure 1:**
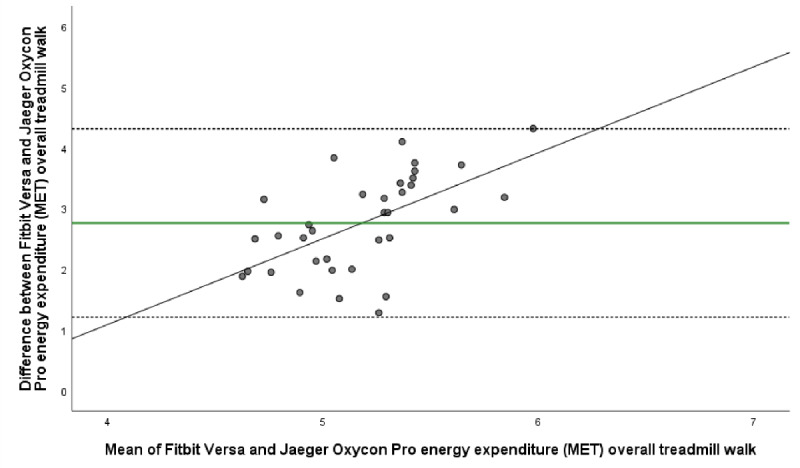
Bland-Altman plot visualizing agreement of energy expenditure (MET) estimated by Fitbit Versa and criterion measurement Jaeger Oxycon Pro during overall treadmill walk. The middle green line shows the mean difference (bias) between devices. The dashed lines indicate upper (+1.96 SD) and lower (–1.96 SD) limits of agreement and the black line represents the regression line illustrating association between estimations.

**Figure 2:**
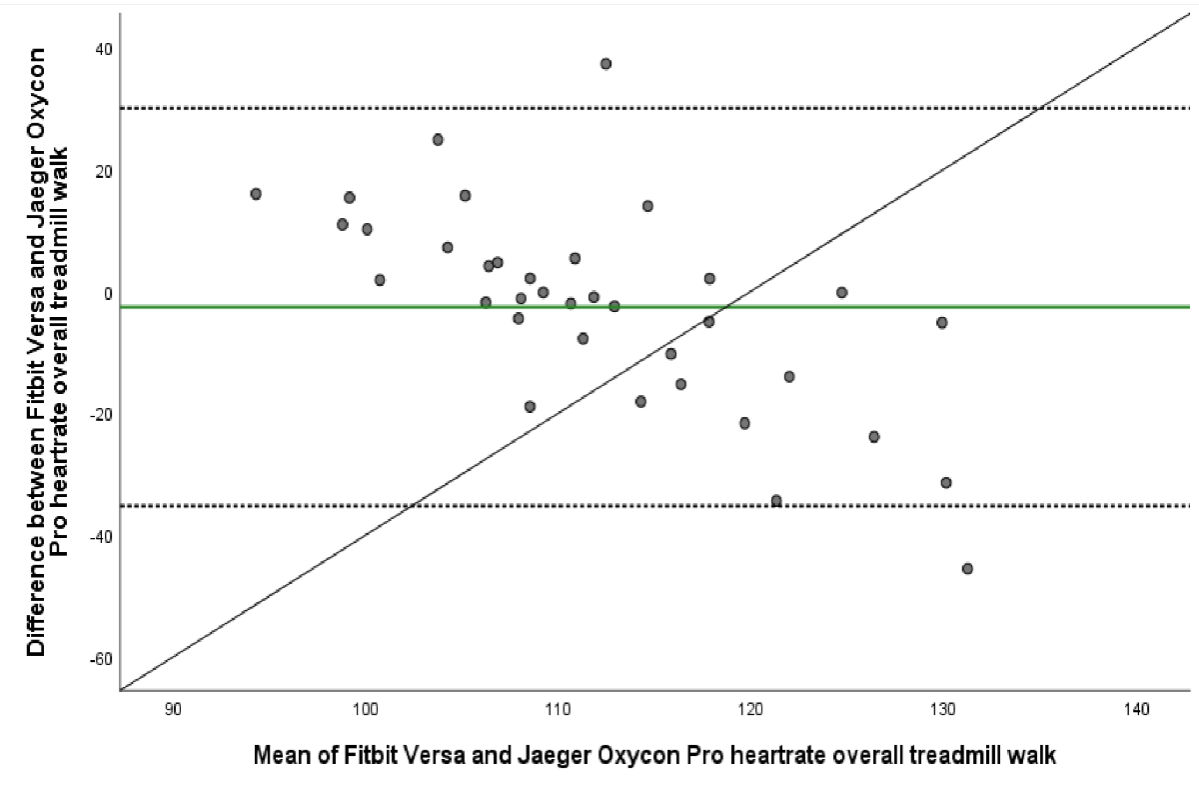
Bland-Altman plot visualizing agreement of heartrate estimated by Fitbit Versa and criterion measurement Jaeger Oxycon Pro during overall treadmill walk. The middle green line shows the mean difference (bias) between devices. The dashed lines indicate upper (+1.96 SD) and lower (–1.96 SD) limits of agreement and the black line represents the regression line illustrating association between estimations.

**Figure 3:**
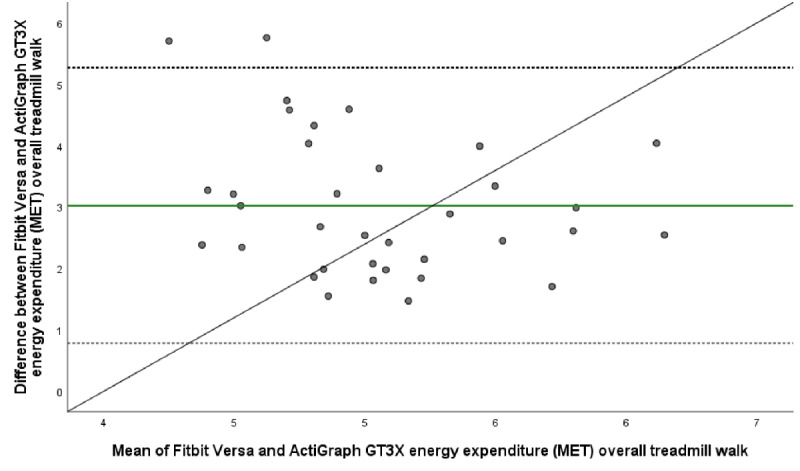
Bland-Altman plot visualizing agreement of energy expenditure (MET) estimated by Fitbit Versa and relative criterion measurement ActiGraph GT3X during overall treadmill walk. The middle green line shows the mean difference (bias) between devices. The dashed lines indicate upper (+1.96 SD) and lower (–1.96 SD) limits of agreement and the black line represents the regression line illustrating association between estimations.

**Figure 4:**
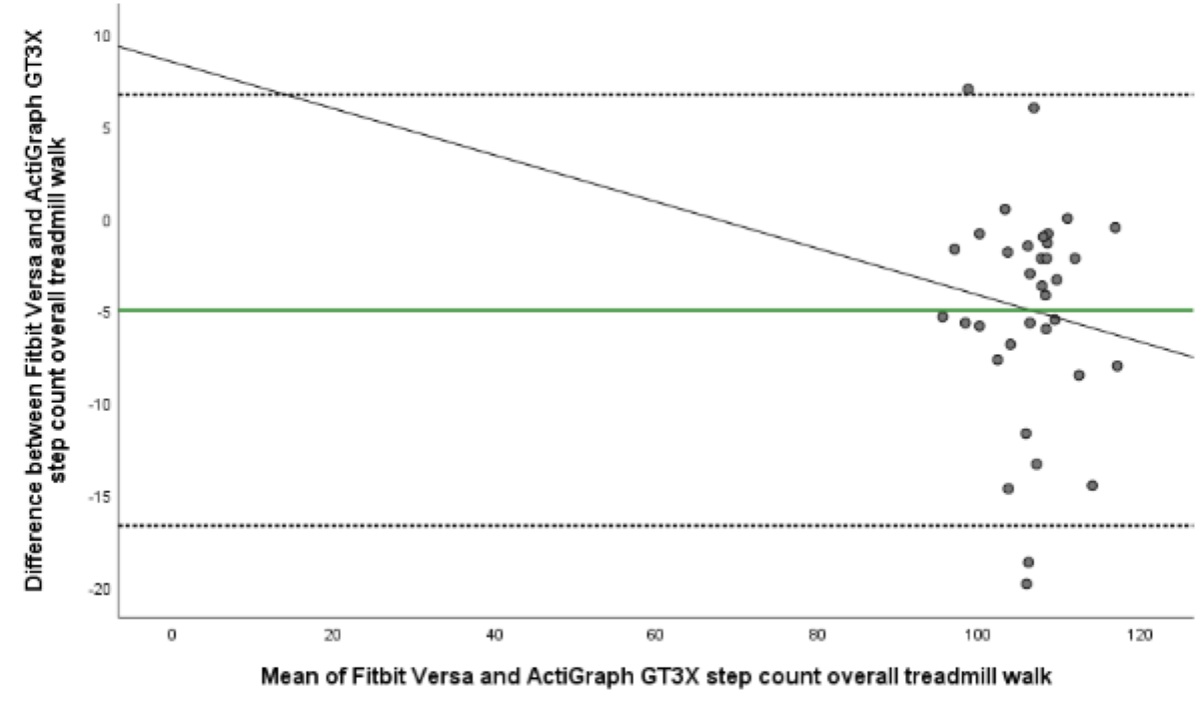
Bland-Altman plot visualizing agreement of step count estimated by Fitbit Versa and relative criterion measurement ActiGraph GT3X during overall treadmill walk. The middle green line shows the mean difference (bias) between devices. The dashed lines indicate upper (+1.96 SD) and lower (–1.96 SD) limits of agreement and the black line represents the regression line illustrating association between estimations.

**Figure 5:**
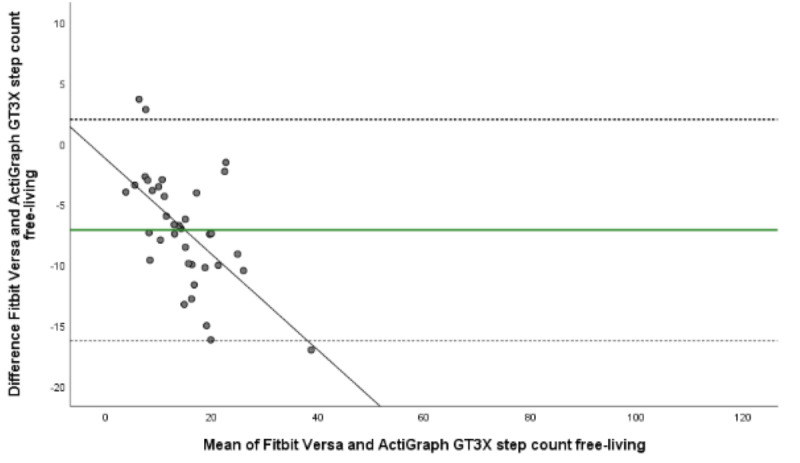
Bland-Altman plot visualizing agreement of step count estimated by Fitbit Versa and relative criterion measurement ActiGraph GT3X during free-living. The middle green line shows the mean difference (bias) between devices. The dashed lines indicate upper (+1.96 SD) and lower (–1.96 SD) limits of agreement and the black line represents the regression line illustrating association between estimations.

The mean energy expenditure, heart rate, and step count of the criterion standard (Jaeger Oxycon Pro), the relative criterion measure (ActiGraph GT3X), and the experimental measure (Fitbit Versa) are presented in Table 3. The ICC (95% CI), mean difference with upper and lower LOA, Spearman correlation, and MAPE for all statistical calculations are presented in Table 4 and Table 5. The Bland-Altman plots for energy expenditure, step count, and heart rate are shown in Figures 1-5.

**Table 3:** Energy expenditure, heart rate, and step count during treadmill walking and in free-living setting.

Measure			Seated rest	Treadmill walk				Free-living setting	
				3.0 km/h	4.5 km/h	6.0 km/h	Overall		
**Energy expenditure**									
	**MET/minute, mean (SD)**								
		Jaeger Oxycon Pro	0.73 (0.17)^a^	2.76 (0.36)	3.50 (0.37)	5.10 (0.42)^b^	3.80 (0.33)^b^		N/A^c^
		ActiGraph GT3X	1.00 (0.00)	1.31 (0.40)	3.91 (1.40)	5.48 (1.07)^b^	3.56 (0.84)^b^		2.53 (0.52)^d^
		Fitbit Versa	1.0 (0.02)	5.73 (0.56)^b^	6.41 (0.58)^a^	7.56 (1.17)^b^	6.56 (0.64)^e^		3.73 (0.86)^d^
	***P* value**								
		Jaeger Oxycon Pro–Fitbit Versa	<.001	<.001	<.001	<.001	<.001		N/A
		ActiGraph GT3X–Fitbit Versa	N/A	<.001	<.001	<.001	<.001		<.001
**Heart rate**									
	**bpm, mean (SD)**								
		Jaeger Oxycon Pro	71.25 (9.82)^a^	99.15 (15.54)^a^	109.61 (14.89)^a^	132.14 (19.48)^b^	113.53 (16.14)^b^		N/A
		Fitbit Versa	72.11(11.55)	101.83 (10.92)^a^	108.67 (8.18)	121.95 (9.63)^b^	110.85 (7.05)^b^		N/A
	***P* value**								
		Jaeger Oxycon Pro–Fitbit Versa	.81	.37	.77	.002	0.34		N/A
**Step count**									
	**Steps/minute**								
		ActiGraph GT3X	N/A	92.70 (8.78)^a^	110.83 (7.08)	124.93 (6.52)^e^	108.61 (6.11)^e^		18.64 (8.51)^d^
		Fitbit Versa	N/A	91.89 (8.75)	106.72 (7.05)	114.20 (11.09)^e^	103.95 (5.62)^e^		11.34 (5.87)^f^
	***P* value**								
		ActiGraph GT3X–Fitbit Versa	N/A	.53	<.001	<.001	<.001		<.001

^a^n=40.

^b^n=39.

^c^N/A: not applicable.

^d^n=38.

^e^n=36.

^f^n=37.

**Table 4:** Comparison between experimental measurement (Fitbit Versa) and the criterion standard (Jaeger Oxycon Pro) in the laboratory setting.

Test and measure			Jaeger Oxycon Pro vs Fitbit Versa (n=41)			
			Energy expenditure–energy expenditure	Heart rate–heart rate	Energy expenditure–heart rate	Energy expenditure–step count
**Seated rest^a^**						
	ICC^b^ (95% CI)		0.003 (–0.16 to 0.20)	0.99 (0.98 to 0.99)	N/A^c^	N/A
	Mean difference (LOA^d^)		0.27 (–0.07 to 0.61)	0.09 (–4.35 to 4.52)	N/A	N/A
	ρ (*P* value)		–0.03 (.86)	0.96 (<.001)	0.27 (.09)	
	MAPE^e^		28.46	2.24	N/A	N/A
**Treadmill walk**						
	**3.0 km/h^f^**					
		ICC (95% CI)	0.01 (–0.04 to 0.04)	0.09 (–0.72 to –0.52)	N/A	N/A
		Mean difference (LOA)	2.97 (1.61 to 4.34)	–2.68 (–39.01 to 33.65)	N/A	N/A
		ρ (*P* value)	–0.14 (.39)	0.24 (.14)	0.10 (.53)	–0.04 (.82)
		MAPE	51.52	11.54	N/A	N/A
	**4.5 km/h^g^**					
		ICC (95% CI)	–0.03 (–0.09 to –0.07)	0.20 (–0.55 to 0.58)	N/A	N/A
		Mean difference (LOA)	2.91 (1.39 to 4.43)	0.75 (–30.73 to 32.22)	N/A	N/A
		ρ (*P* value)	–0.31 (.05)	0.16 (.33)	–0.11 (.51)	–0.19 (.24)
		MAPE	44.80	10.19	N/A	N/A
	**6.0 km/h^h^**					
		ICC (95% CI)	–0.05 (–0.19 to –0.16)	0.40 (–0.09 to 0.68)	N/A	N/A
		Mean difference (LOA)	2.46 (–0.11 to 5.03)	10.19 (–25.56 to 45.95)	N/A	N/A
		ρ (*P* value)	–0.11 (.51)	0.44 (.01)	0.17 (.34)	0.01 (.97)
		MAPE	31.59	12.10	N/A	N/A
	**Overall speeds^i^**					
		ICC (95 % CI)	–0.03 (–0.08 to 0.08)	0.19 (–0.58 to 0.59)	N/A	N/A
		Mean difference (LOA)	2.76 (1.21 to 4.31)	–2.68 (–35.30 to 29.95)	N/A	N/A
		ρ (*P* value)	–0.22 (.20)	0.23 (.17)	–0.05 (.75)	0.07 (.71)
		MAPE	35.39	10.56	N/A	N/A

^a^n=40.

^b^ICC: intraclass correlation.

^c^N/A: not applicable.

^d^LOA: limits of agreement.

^e^MAPE: mean absolute percent error.

^f^n=39 for energy expenditure–energy expenditure; n=40 for heart rate–heart rate and energy expenditure–heart rate.

^g^n=40 for energy expenditure–energy expenditure and heart rate–heart rate.

^h^n=36 for energy expenditure–energy expenditure; heart rate–heart rate, and energy expenditure–heart rate; n=39 for energy expenditure–step count.

^i^n=35 for energy expenditure–energy expenditure and energy expenditure–step count; n=36 for energy expenditure–heart rate and heart rate–heart rate.

**Table 5:** Comparison between the experimental measurement (Fitbit Versa) and the relative criterion measurement (ActiGraph GT3X) in both settings (laboratory and free-living).

Test and measure			ActiGraph GT3X vs Fitbit Versa (n=41)			
			Energy expenditure–energy expenditure	Step count–step count	Energy expenditure (ActiGraph GT3X)–step count (Fitbit Versa)	Step count (ActiGraph GT3X)–energy expenditure (Fitbit Versa)
**Seated rest**						
		ICC^a^ (95% CI)	N/A^b^	N/A	N/A	N/A
		Mean difference (LOA^c^)	0.00 (–0.33 to 0.34)	N/A	N/A	N/A
		ρ (*P* value)	N/A	N/A	N/A	N/A
		MAPE^d^	0.36	N/A	N/A	N/A
**Treadmill walk**						
	**3.0 km/h^e^**					
		ICC (95% CI)	–0.01 (–0.03 to 0.04)	0.71 (0.44 to 0.84)	N/A	N/A
		Mean difference (LOA)	4.43 (2.92 to 5.94)	0.84 (–15.73 to 17.40)	N/A	N/A
		ρ (*P* value)	–0.26 (.11)	0.66 (<.001)	–0.08 (.60)	0.42 (.01)
		MAPE	76.86	5.39	N/A	N/A
	**4.5 km/h^f^**					
		ICC (95% CI)	0.02 (–0.12 to 0.21)	0.69 (0.29 to 0.85)	N/A	N/A
		Mean difference (LOA)	2.55 (–0.36 to 5.45)	4.11 (–8.13 to 16.35)	N/A	N/A
		ρ (*P* value)	0.11 (.51)	0.66 (<.001)	0.07 (.66)	0.60 (<.001)
		MAPE	39.44	4.98	N/A	N/A
	**6.0 km/h^g^**					
		ICC (95% CI)	–0.14 (–0.50 to 0.24)	0.05 (–0.51 to 0.35)	N/A	N/A
		Mean difference (LOA)	2.08 (–1.30 to 5.45)	10.79 (–15.02 to 36.61)	N/A	N/A
		ρ (*P* value)	–0.07 (.67)	0.09 (.60)	0.23 (.18)	0.37 (.03)
		MAPE	25.01	11.15	N/A	N/A
	**Overall speeds^h^**					
		ICC (95% CI)	–0.04 (–0.13 to 0.12)	0.60 (0.03 to 0.82)	N/A	N/A
		Mean difference (LOA)	3.02 (0.78 to 5.26)	–4.98 (–16.68 to 6.71)	N/A	N/A
		ρ (*P* value)	–0.10 (.56)	0.51 (<.002)	0.28 (.11)	0.31 (.07)
		MAPE	39.41	5.41	N/A	N/A
**Free-living**						
	**Overall days^i^**					
		ICC (95% CI)	0.46 (–0.16 to 0.80)	0.70 (–0.21 to 0.91)	N/A	N/A
		Mean difference (LOA)	1.20 (0.17 to 2.24)	–7.12 (–16.25 to 2.00)	N/A	N/A
		ρ (*P* value)	0.79 (<.001)	0.87 (<.001)	0.41 (.01)	0.55 (<.001)
		MAPE	31.11	82.45	N/A	N/A

^a^ICC: intraclass correlation.

^b^N/A: not applicable.

^c^LOA: limits of agreement.

^d^MAPE: mean absolute percent error.

^e^n=39 for energy expenditure–energy expenditure; n=40 for step count–step count, n=38 for energy expenditure–step count.

^f^n=40 for energy expenditure–energy expenditure and step count–energy expenditure.

^g^n=36 for energy expenditure–energy expenditure; n=34 for step count–step count; n=35 for energy expenditure–step count and step count–energy expenditure.

^h^n=35 for energy expenditure–energy expenditure; n=34 for step count–step count; n=35 for energy expenditure–step count; n=34 for step count–energy expenditure.

^i^n=38 for energy expenditure–energy expenditure and step count–energy expenditure; n=37 for step count–step count and energy expenditure–step count.

### Fitbit Versa versus Jaeger Oxycon Pro

In the laboratory setting we found that Fitbit Versa showed poor agreement of estimated energy expenditure with corresponding estimations by Jaeger Oxycon Pro in the overall treadmill walk (ICC –0.03, 95% CI –0.08 to 0.08). There were also significant systematic differences between estimations in all treadmill speeds as well as in the overall treadmill walk (*P≤*.001). In addition, the Bland-Altman plot showed a broad range for energy expenditure estimation, also indicated the overestimation, with a mean difference of 2.76 MET, LOA 1.21 to 4.31 for overall speeds (Table 4, Figure 1). A narrow mean difference was found during rest, 0.27 MET, LOA –0.07 to 0.61. In addition, the correlation of energy expenditure estimated by Fitbit Versa and Jaeger Oxycon Pro was weak at all measured timepoints. Overall treadmill speed MAPE for energy expenditure was 35.39 and ranged from 31.59 at 6 km/h to 51.52 at 3.0 km/h.

There was poor agreement between Fitbit Versa’s estimation of heart rate compared to Jaeger Oxycon Pro at overall treadmill (ICC 0.19, 95% CI –0.58 to 0.59). At the specific treadmill speeds ICC ranged from poor (ICC 0.09, 95% CI –0.72 to 0.52) at 3.0 km/h to fair (ICC 0.40, 95% CI –0.09 to 0.68) at the final treadmill speed (6 km/h). However, agreement of estimations was excellent (ICC 0.99, 95% CI 0.98 to 0.99) and correlation very strong (ρ=.96, *P≤*.001) during seated rest. ANOVA results showed no systematic differences between estimations of heart rate during rest (*P*=.81), at 3 km/h (*P*=.37), at 4.5 km/h (*P*=.77), or during the overall treadmill walk (*P*=.34). This was also confirmed by the Bland-Altman plot; the mean difference of heart rate estimation during the overall treadmill walk were –2.68 bpm, LOA –35.30 to 29.95 bpm. It ranged from –2.68 bpm, LOA –39.01 to 33.65 at 3.0 km/h to a broader range, 10.19 bpm, LOA –25.45 to 46.57 at 6 km/h (Table 4, Figure 2). Corresponding MAPE ranged from 2.24 at seated rest to 12.10 at 6 km/h, with the overall treadmill walk at 10.51.

We found only weak correlations between energy expenditure by Jaeger Oxycon Pro and heart rate by Fitbit Versa, and between energy expenditure by Jaeger Oxycon Pro and step count by Fitbit Versa, during both seated rest and during all treadmill speeds (Table 4).

In accordance with findings of poor agreement between Fitbit Versa and the criterion measurement’s (Jaeger Oxycon Pro) estimations of energy expenditure, we also found poor agreement between corresponding estimations by Fitbit Versa and the relative criterion measurement ActiGraph GT3X, at all treadmill speeds (Table 4). For the overall treadmill walk, the agreement was poor (ICC –0.04, 95% CI –0.13 to 0.12) as it also was at specific treadmill speeds (Table 5).

### Fitbit Versa versus ActiGraph GT3X

Due to zero variation in data, ICC calculations of energy expenditure estimated by ActiGraph GT3X and Fitbit Versa during seated rest were not possible to perform. The Bland-Altman plot provided a mean difference of 0.00 MET, LOA –0.33 to 0.34 to for seated rest indicating a high agreement in estimations of heart rate between the devices (Table 4). Also, there were minimal individual differences between measurements during rest (MAPE 0.36) but greater differences (MAPE 76.86) at 3.0 km/h, however they decreased as treadmill speed increased (MAPE 39.44 at 4.5 km/h, MAPE 25.01 at 6 km/h) (Table 5).

Findings suggest a fair agreement (ICC 0.54, 95% CI 0.02 to 0.78) and a strong significant correlation (ρ=0.51, *P≤*.001) of step count estimations by Fitbit Versa and ActiGraph GT3X at the overall treadmill level (Table 5, Figure 3). At specific treadmill speeds, the agreement was good at both 3.0 km/h (ICC 0.71, 95% CI 0.44 to 0.84) and at 4.5 km/h (ICC 0.69, 95% CI 0.29 to 0.85), but decreased at 6 km/h (ICC 0.05, 95% CI –0.51 to 0.35) (Table 5).

There was fair and significant correlation in step count between devices in 2 out of 3 treadmill speeds (3.0 km/h, 4.5 km/h) and the overall treadmill walk (ρ=0.51, *P≤*.001). The ANOVA results were significant for the overall treadmill walk and at the 2 higher treadmill speeds (*P≤*.001) while the Bland-Altman plots showed a mean difference at the overall speed by –4.98 steps, LOA –16.68 to 6.71 (Table 5, Figure 4). MAPE ranged from 5.39 at 3.0 km/h to 11.15 at 6 km/h, with 5.41 for the overall treadmill walk (Table 5). The correlation between ActiGraph GT3X estimations of energy expenditure and Fitbit estimations of step count were weak for the treadmill walk in the laboratory setting. However, the correlation between ActiGraph GT3X estimations of step count and Fitbit estimations of energy expenditure were significant and fair for the slowest (ρ=0.42, *P*=.01) and fastest (ρ=0.37, *P*=.37) treadmill speed. Moderate and significant correlation (ρ=0.60, *P≤*.001) was found at 4.5 km/h (Table 5).

In the free-living setting, we found fair agreement between Fitbit Versa and ActiGraph GT3X’s estimations of energy expenditure (ICC 0.46, 95% CI –0.16 to 0.80), and a significant and strong correlation (ρ=0.79, *P≤*.001). ANOVA results show no systematic differences between estimations (*P≤*.001), which is confirmed by the Bland-Altman plot mean difference by 1.20 MET, LOA 0.17 to 2.24 MET and MAPE 31.11 (Table 5). The agreement between Fitbit Versa and ActiGraph GT3X’s estimations of step count were good (ICC 0.70, 95% CI –0.21 to 0.91) and the correlation between estimations was strong (ρ=0.87, *P≤*.001). ANOVA results showed no systematic differences between step count estimations (*P≤*.001). Bland-Altman plot showed a mean difference with –7.12 steps, LOA –16.25 to 2.00 confirming an agreement (Figure 5). MAPE, on the other hand, was 82.45, indicating great individual bias (Table 5).

The correlation between ActiGraph GT3X estimations of energy expenditure and Fitbit Versa estimations of step count were significant and fair (ρ=0.41, *P*=.01). A corresponding association was found (ρ=0.55, *P≤*.001) between ActiGraph GT3X’s step count, and Fitbit Versa’s estimation of energy expenditure (Table 5).

## Discussion

### Principal Findings

To our knowledge, this is the first study that has evaluated criterion validity of Fitbit Versa’s estimations of energy expenditure, step count, and heart rate for patients with chronic pain. Evaluations of criterion validity wrist-worn outputs of energy expenditure, heart rate, and step count is essential before any clinical application may be implemented [39]. Poor agreement (ICC, mean difference and LOA, MAPE) as well as poor correlation were found between the criterion measurement (Jaeger Oxycon Pro) and the experimental measurement (Fitbit Versa) regarding energy expenditure for the overall treadmill walk as well as the 3 specific treadmill speeds (Table 4). However, good agreement and fair correlation emerged between estimations of step count by Fitbit Versa and ActiGraph GT3X for the majority of the treadmill walk as well as the overall treadmill walk (Table 5). Good agreement and correlation were shown for the estimation of heart rate during seated rest as well, but this decreased during all treadmill speeds.

### Comparison With Previous Studies

Findings suggest that Fitbit Versa systematically overestimated energy expenditure across the full range of the testing protocol when compared to both the criterion (Jaeger Oxycon Pro) and the relative criterion measurement (ActiGraph GT3X). A strict comparison of our study findings with other research within this population was not possible due to the lack of such studies. On the other hand, studies have been conducted aiming to evaluate criterion validity in wrist-worn activity trackers among an elderly population [56] as well as among populations suffering from chronic cardiac conditions [57,58]. These reports also suggest an overestimation of energy expenditure. Herkert et al [57] studied the accuracy of Fitbit Charge 2 among patients with chronic heart conditions and compared estimations of energy expenditure with indirect calorimetry (Oxycon Mobile) during several household activities and a treadmill walk (4.0 km/h, 5.5 km/h, 4.0 km/h + 5% slope). While their findings are not strictly applicable to our sample, both samples included patients with physically impairments and findings suggested a clear overestimation of energy expenditure [57].

The results of other studies [19,24], conducted primarily among healthy participants and examining the validity of other Fitbit models’ ability to estimate energy expenditure, contradict our results—certain Fitbit models (Fitbit, Fitbit Ultra/Fitbit Zio, Fitbit Flex) underestimated energy expenditure and step count when data were compared to the criterion measurements. Furthermore, we found good agreement between step count by Fitbit Versa and ActiGraph GT3X for the first 2 treadmill speeds, as well as fair agreement for the overall treadmill test, but agreement decreased at 6 km/h. In the free-living setting, we found good agreement and excellent correlation between step count estimation by Fitbit Versa and ActiGraph GT3X. This corresponds to the findings of a study [59] that compared step count estimation of healthy participants in a free-living setting using a Fitbit device (Fitbit One) and ActiGraph GT3X, and reported excellent agreement between the 2 measurements.

The overestimation of energy expenditure found in our study could be explained by the proprietary algorithms applied by Fitbit, which are not tailored to specific populations. In the specific population related to this study, altered movement patterns is indicated due to changed motor control and kinematics as well as due to a fear of pain causing a protective avoidance in movement and activity [60-62]. Fitbit’s estimation of energy expenditure is based on both body composition metrics as well as (if available in the device) estimated heart rate [33,34], which requires a valid heart rate measurement. Our findings indicate a poor to fair criterion validity in estimations of heart rate for all treadmill speeds, which is consistent with previous studies [28,63]. Another important factor that may have influenced our findings is the placement of devices on the body. Our experimental device was placed on participants’ wrists according to the manufacturer’s instructions, and the relative criterion measurement device were placed around participants’ waists, near their right hip (also according to the manufacturer’s instructions). Feehan with colleges concludes that placing devices on the wrist generally leads to an overestimation of energy expenditure which may be explained by the waist sensor being placed closer to the center of the body [23].

### Strengths and Limitations

Data collection was performed in both a laboratory, limiting many confounding variables, and in a natural free-living context, increasing the ecological validity, which is in line with current recommendations for validity studies examining wearable monitors for physical activity [39,40]. In this study, conventional statistics were applied in order to conduct a comprehensive evaluation of the criterion validity and report findings in a standardized manner [54]. Equivalence testing is also recommended as it provides both a risk evaluation of measurement agreement and zones of equivalence between estimations is established by consensus [54]. This method was not applied in this study, which may be a limitation, as knowledge of statistically significant risk of misclassified physical activity–level would certainly contribute to the interpretation of the results. The sample size was in accordance with the initial sample size calculation and equivalent to corresponding research studies [20,22,57,59,64]. Furthermore, when conducting validation studies, guidelines state that one should perform measurements in a large range of physical activity intensities [39]. In this study, treadmill speed was set to a maximum of 6 km/h which may be interpreted as a low intensity; however, we suspected that a higher speed could have been problematic for some participants. The fact that 5 of the 41 participants were unable to complete the treadmill walk at 6 km/h indicates that a higher treadmill speed could have resulted in a higher number of discontinuations. A possible source of bias in validation studies is pharmacological use of beta blockers because it affects heart rate and possibly biases evaluation of physical activity intensity level. In this study, only 2 participants (<5%) reported taking beta blockers within the last 24 hours which may have affected participants perceived exertion during treadmill use. However, we estimate this having a very little impact on our results.

Our sample included 76% women (31/41), which is in line with other studies describing people with chronic pain in Sweden [65,66] and other countries [67]. Patients in our sample had lived with pain for a shorter time than what is described in other studies of this population [68,69], and they rated their pain severity at baseline as equal to what have been reported in another study [70] describing patients participating in primary care management of low back pain patients. In our study, 37% of participants reported an exercise level of >90 minutes/week and 49% reported being physically active >150 minutes/week. A previous study [71] reported that that 38% of participating men and 37% of participating women with chronic pain were physically active >60 minutes/week. Since almost half of our participants report reaching the recommended weekly amounts of physical activity, it seems that our sample is slightly more physically active than the population with chronic pain, in general. In all, we believe that the external validity can be extended to the major group of individuals with chronic pain seeking care in primary and specialist care.

### Conclusions

This study provides new knowledge on the criterion validity of Fitbit Versa’s estimations of energy expenditure, heart rate, and step count in patients with chronic pain. Findings show that Fitbit Versa overestimates energy expenditure when compared to criterion estimations in a controlled laboratory setting as well as in free-living settings, which needs to be considered when used clinically for patients with chronic pain. Step count measured from the wrist, however, seems to provide a valid estimation, suggesting that future guidelines should include this variable in this major patient group. Findings may contribute to the solicited documentation of estimation properties of wrist-worn activity tracking devices within specific patient groups and may therefore guide future application in further clinical research.

## Abbreviations

ANOVA: analysis of variance
ICC: intraclass correlation coefficient
LOA: limits of agreement
MAPE: mean absolute percent error
MVPA: moderate-to-vigorous physical activity
